# Enterohemorrhagic *Escherichia coli* O157, Kinshasa

**DOI:** 10.3201/eid1005.031034

**Published:** 2004-05

**Authors:** Louis Koyange, Gaelle Ollivier, Jean-Jacques Muyembe, Benoit Kebela, Malika Gouali, Yves Germani

**Affiliations:** * Institut National de la Recherche Biomédicale, Kinshasa Gombe, Democratic Republic of Congo; † Ambassade de France, Kinshasa Gombe, Democratic Republic of Congo; ‡ Ministère de la Santé, Kinshasa Gombe, Democratic Republic of Congo; § Institut Pasteur de Bangui, Bangui, Central African Republic; ¶ Institut Pasteur, Paris, France

**Keywords:** hemorrhagic colitis, *Escherichia coli* O157, diarrhea, Kinshasa

**To the Editor:** During the rainy season, from April to September 2003, 463 children ≤15 years of age (median 10 months) with severe diarrhea were admitted to the Pediatric Hospital of Kalembelembe in Kinshasa, the capital of the Democratic Republic of Congo. The population of the outbreak area was approximately one million.

Several children with bloody diarrhea without fever were treated. They came from six districts of Kinshasa (Bumbu, Selembao, Makala, Kimbanseke, Masina, and Ndjili). Abdominal cramps, nausea, vomiting, and dehydration were uncommon. The duration of illness ranged from 5 days to 2 weeks. Available antiparasitic drugs, trimethoprim-sulfamethoxazole, and ampicillin showed no effect against the illness. Fifty-six infants died between June and July. Symptoms of hemolytic-uremic syndrome developed in most of them.

Stool samples from 32 patients were screened for parasites, enteropathogenic bacteria, rotavirus, and adenovirus. Three samples were positive for rotavirus. In contrast, all stool cultures were positive for *Escherichia coli* which always grew as pure cultures on purple bromocresol agar, a nonselective medium containing lactose. The *E. coli* isolates appeared sorbitol negative when tested on MacConkey sorbitol; they were agglutinated by O157 and H7 antisera (Difco Laboratories, Detroit, MI) and lacked expression of β-glucuronidase. All *E. coli* isolates were sent to the Pasteur Institute in Bangui, Central African Republic, for further characterization. Polymerase chain reaction allowed detection of Shiga-like toxin *slt-1* and *slt-2* genes (*1*,*2*) in isolates from all patients. The Vero cell assay phenotypically confirmed cytotoxicity of these isolates, with most of them being seroneutralized by rabbit antisera against Shiga toxin (*3*). Thus, all *E. coli* isolates responded to the definition of enterohemorrhagic *E. coli*.

Before 2003, sporadic infections or outbreaks caused by enterohemorrhagic *E. coli* were not reported as a cause of bloody diarrhea in the Democratic Republic of Congo. A case-control study could not be performed because of political unrest in Kinshasa. Although reported outbreaks of *E. coli* O157 in sub-Saharan Africa have been few to date, available information indicates that the pathogen has wide geographic distribution. *E. coli* O157–related diarrhea outbreaks that occurred before 2003 have been reported in South Africa, Swaziland (*4*), and Malawi (*5*) in 1992; Central African Republic (*6*) and Kenya (*7*) in 1996; Cameroon in 1998 (*8*); and Nigeria (*9*) and Ivory Coast (*10*) in 2000. In the Central African Republic and in Zémio, a small village located on the Democratic Republic of Congo border, outbreaks of bloody diarrhea in 1996 were attributed to *E. coli* O157 from molecular test results (*6*).

Since 2001, an increasing number of cases of acute bloody diarrhea have been reported in Kinshasa between June and August. During this 2003 outbreak, an investigation could not be conducted; possible routes of transmission would include person-to-person contact related to lack of hygiene, and contaminated food and water.

In 1996 in the Central African Republic and in 1998 in Cameroon, the major contributing factors of the *E. coli* O157 outbreak were consumption of smoked zebu meat and contaminated drinking water. Studies of *E. coli* O157 carriage rates among livestock, food, and environment in this central African area might be useful in assessing the potential for future outbreaks.

Hemolytic-uremic syndrome occurs in approximately 8% of children and an unknown proportion of adults infected with *E. coli* O157 and can be fatal without hemodialysis. The high death rate of infants during this outbreak was linked to the lack of treatment (mainly hemodialysis) at the beginning of the epidemic. Obviously, more work is needed to better define the incidence and epidemiology of *E. coli–*associated diarrhea in the Democratic Republic of Congo so that optimal recommendations for preventing and managing illness can be developed.
